# Supplementary material for: The role of C-O-H-F-Cl fluids in the making of Earth’s continental roots
Source: Nat Commun. 2025 Aug 22;16:7842. doi: 10.1038/s41467-025-62888-3 (PMC12373800; doi:10.1038/s41467-025-62888-3)
Supplement: Supplementary file 1 — Supplementary Information [file 41467_2025_62888_MOESM1_ESM.pdf]

The role of C-O-H-F-Cl fluids in the making of Earth's continental roots

Gibson, S.A.[1*] Jackson, C.J. [1], Crosby, J.C. [1] & Day, J.A.F. [1]

[1]Dept of Earth Sciences, University of Cambridge, Downing St, Cambridge, UK. CB2 3EQ.

[*]Corresponding author: sally@esc.cam.ac.uk

Supplementary materials

Textural relationships in mantle peridotites provide important information on their formation. Orthopyroxene-rich garnet harzburgites from the Kaapvaal craton are typically coarse-grained and sometimes contain cm-scale veins or segregations of orthopyroxene (Figure S1).

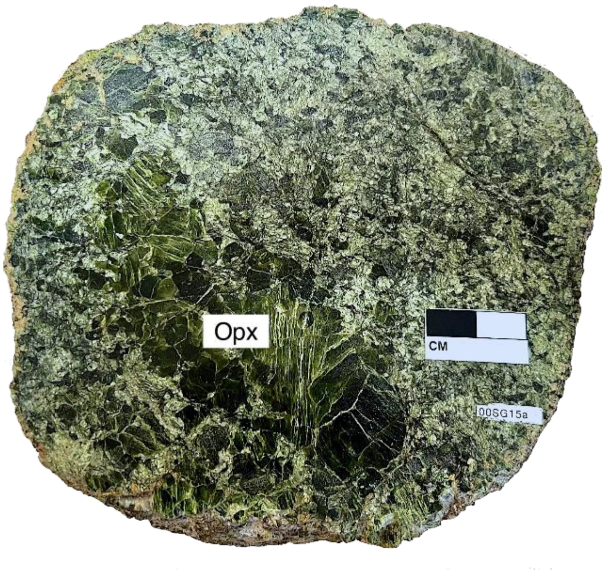

Figure S1. Coarse grained vein of orthopyroxene traversing a harzburgite (00SG15a) from the Kaapvaal craton of southern Africa.

In thin section, the orthopyroxenes can often be seen to enclose, and therefore post-date, the olivines (Figure S2) in the orthopyroxene-rich garnet harzburgites. This is especially apparent in the diamond-bearing sample BD2125, which was entrained by the Late Cretaceous Mothae kimberlite (Lesotho) on the southeastern margin of the Kaapvaal craton (Figure S3). This mantle peridotite is highly enriched in orthopyroxene (39 %) and depleted in olivine (52 %), compared to the amounts predicted by thermodynamical models for residues of mantle melting (Figure 3b). Thermobarometry indicate that the xenolith equilibrated at 1055°C and 4.4 GPa[3].

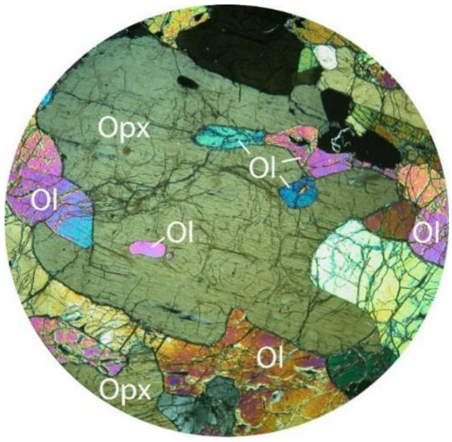

Figure S2. Photomicrograph of a rare diamond-bearing garnet harzburgite (BD2125)[4] showing the textural relationship between olivine (Ol) and orthopyroxene (Opx). The image is taken in cross polarised light and the field of view is 1 cm.

Our study primarily focuses on mantle peridotites from the Kaapvaal craton, entrained by kimberlites from Bultfontein and Mothae, and is supplemented by additional samples from the Tanzania craton at Lashaine (Figure S3).

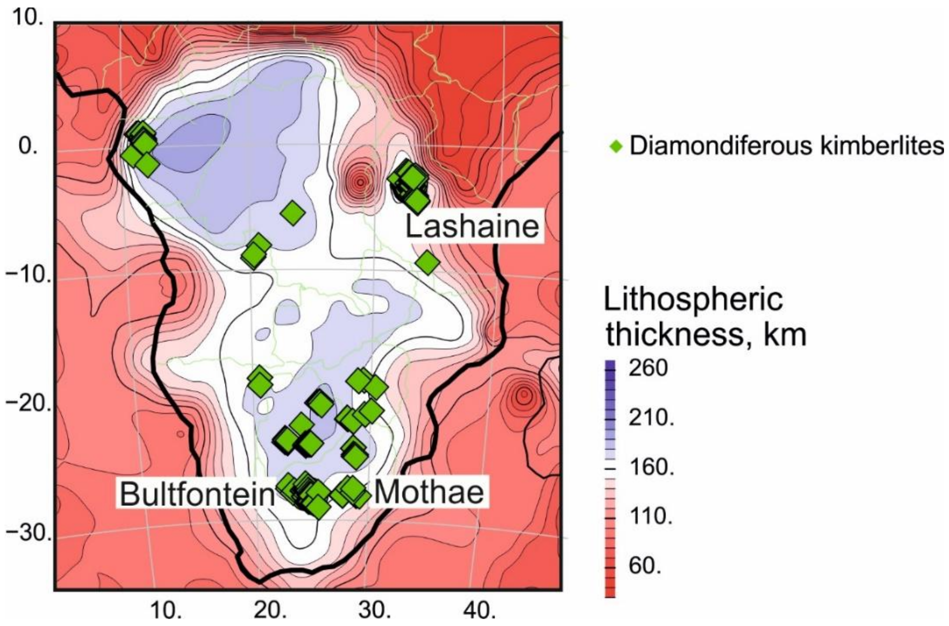

Figure S3. Location of mantle peridotites analysed in this study. The locations of diamondiferous kimberlites and contours illustrating the seismic lithospheric thickness are from refs[1,2].

The orthopyroxene-rich harzburgites contain olivine with similar Fo contents ($\bar{x}$=93) to those found in other garnet harzburgites ($\bar{x}$=92.79; Figure S4). The most magnesian are found in the spinel harzburgites ($\bar{x}$=94.0) and dunite (94.8) and the most Fe-rich occur in garnet lherzolites ($\bar{x}$=91.02).

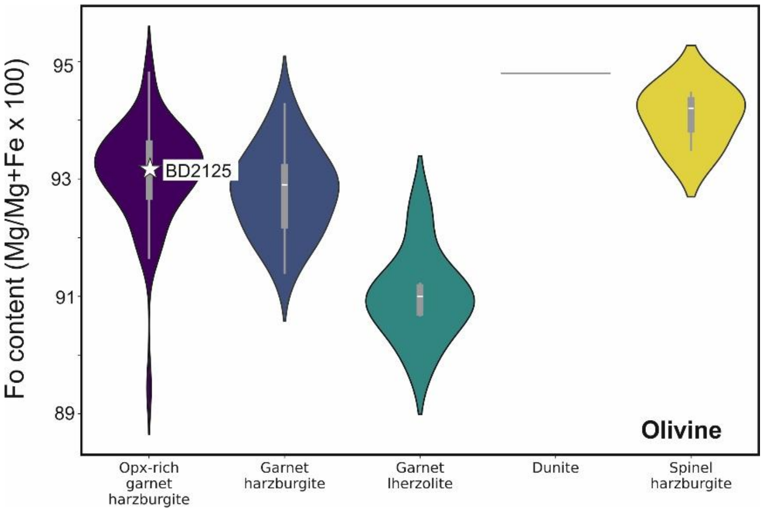

Figure S4. Violin plot illustrating the variation of Fo contents (Mg/Mg+Fe x 100) in olivine from different types of mantle peridotites found in the Kaapvaal, Tanzania and Siberia cratons previously analysed for volatiles (H_2O). The box shows the quartiles of the dataset while the whiskers extend to show the rest of the distribution. The median is shown by the grey line. Data are from this work and refs [3,5–11].

Table S1. Summary of the data shown in Figure S4.

Lithology	Mean Fo content	Number of samples for each lithology
Opx-rich garnet harzburgite	93.09	49
Garnet harzburgite	92.79	19
Garnet lherzolite	91.02	7
Dunite	94.8	1
Spinel harzburgite	94.06	3

Our new data for H_2O in Kaapvaal mantle olivine and pyroxenes are consistent with previous analyses for the Kaapvaal craton[5]. Our F data are the first set of analyses for cratonic mantle (Figure S6). Previous studies involving F in mantle peridotites have been focused on off-craton mantle [12,13].

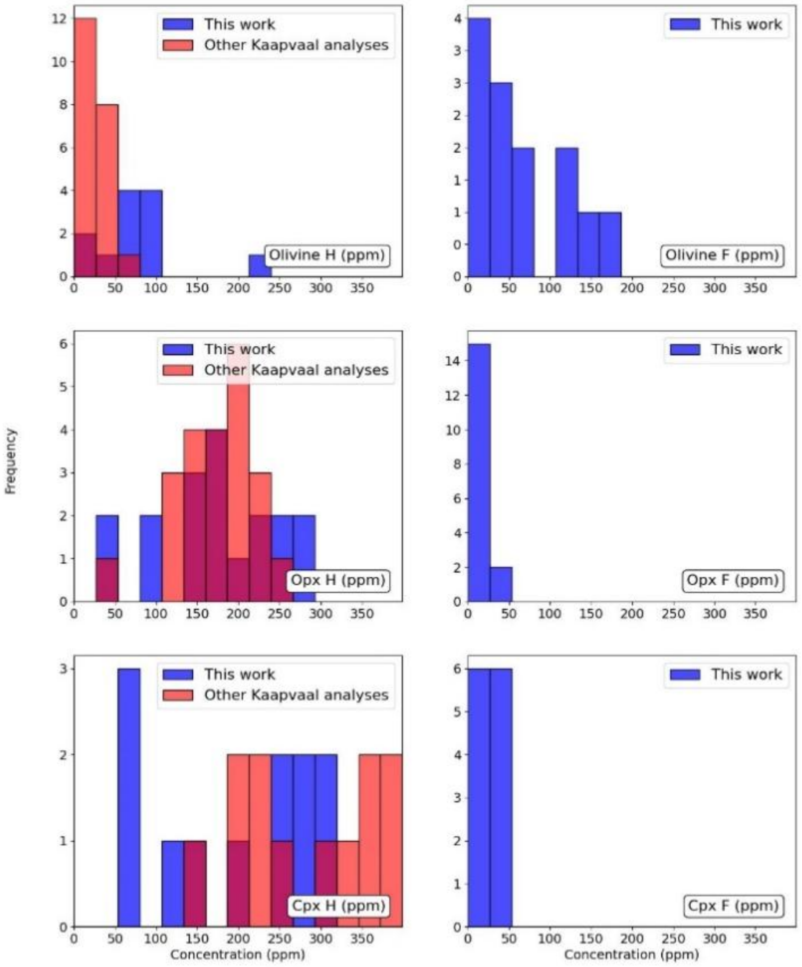

Figure S5. Comparison of SIMS analyses from this work with previous data for the Kaapvaal craton peridotites[5].

Table S2. Comparison of determinations of H_2O (in ug/g) by SIMS and FTIR (based on the calibration of ref[14]) from mineral grains in the same mantle peridotites from the Kaapvaal craton.

	Olivine		Orthopyroxene	
	This work	Heckel (2023)[15]	This work	Heckel (2023)[15]
	SIMS (Edinburgh)	FTIR (Bayreuth)	SIMS (Edinburgh)	FTIR (Bayreuth)
BD2128	22	30	191	155
BD2170		44	183	180

The pyroxenes are the main host of H_2O in the Kaapvaal peridotites (Figure S5). In contrast, olivine tends to be the main host of F. The variability in concentration of H_2O and F within each lithology is quite large but there are important correlations of F with modal mineralogy within the orthopyroxene-rich harzburgites (Figure 7). Also, there are significant inter lithological variations in bulk xenolith H_2O/Ce and Pr/F ratios (Figure 5).

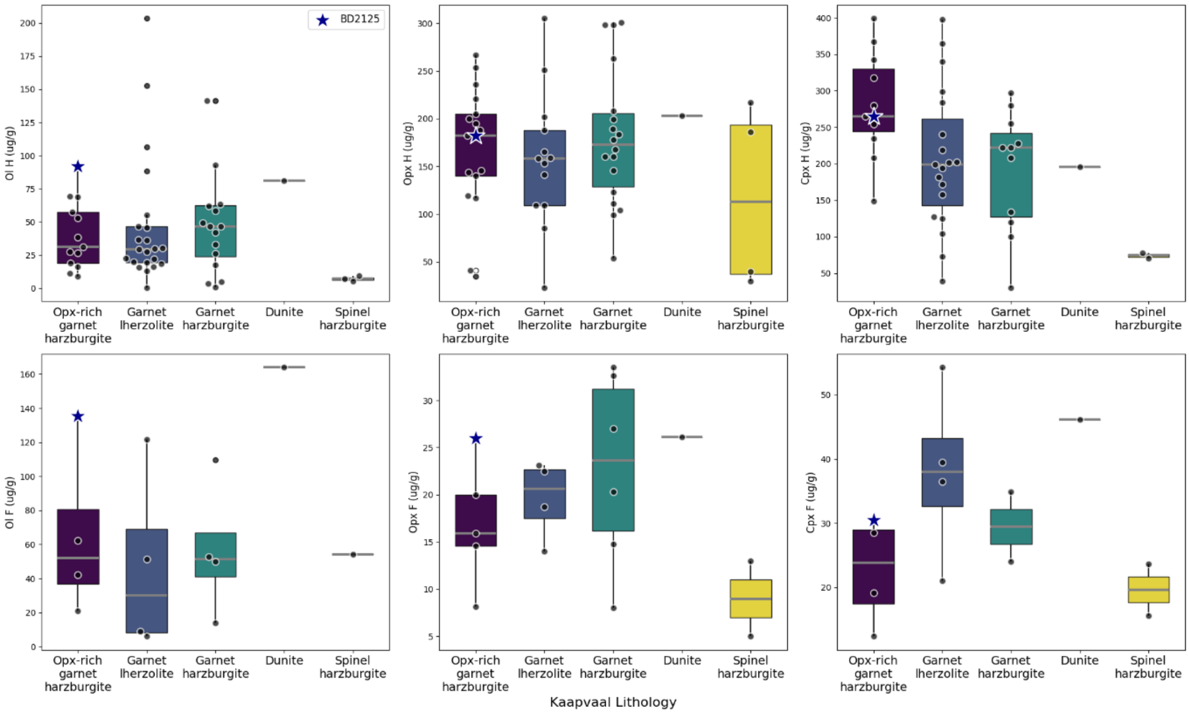

Figure S6. Box and whisker plots showing variations in H_2O and F contents in olivine and pyroxenes in different types of mantle peridotites from the Kaapvaal craton. Data are from this work and Peslier et al (2012)[5] .The boxes define the edge of the 1st and 3rd quartile ranges, the grey line inside the box is the median value and the whiskers represent the range of the data. A summary of the statistical analyses of bulk volatile data are available in the Supplementary Data 6.

The sinusoidal MORB source normalised rare-earth patterns of the garnets in the orthopyroxene-rich garnet harzburgites with minima at Er-Tm resemble some of the inclusions in diamonds from Finsch Mine on the Kaapvaal craton (Figure S7). While it is not clear if the inclusions formed before or during diamond formation their resemblance to those in the orthopyroxene-rich garnet harzburgites suggest a similar origin.

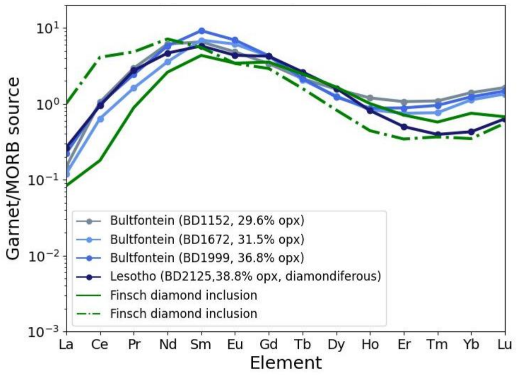

Figure S7. Rare-earth-element patterns of garnets in orthopyroxene-rich harzburgites [3]. Finsch diamond inclusion data are from [16] and normalisation factors are from[17] .

An interesting observation is that the diamondiferous orthopyroxene-rich garnet harzburgite (BD2125) contains phlogopite and this has higher Cl (0.17 µg/g) but lower Na_2O (0.07 wt. %) and TiO_2 (0.12 wt. %) contents than found in other mantle lithologies (<0.05 µg/g Cl, Na_2O= 0.11 to 0.24, TiO_2=0.49 to 1.0 wt.%). This provides important additional information on the alkalinity and volatile composition of the infiltrating fluids involved in the formation of excess orthopyroxene, i.e. they were rich in K, Na, C, H, F and Cl.

The large amounts of melting involved in the initial formation of cratonic mantle deplete the concentrations of most incompatible trace elements (Figure S8). Rare-earth element inversion models show that the heavy rare-earth concentrations (Dy, Ho, Er, Tm and Lu) can be explained by ~40 % adiabatic decompression melting but more incompatible trace elements require enrichment of this residue.

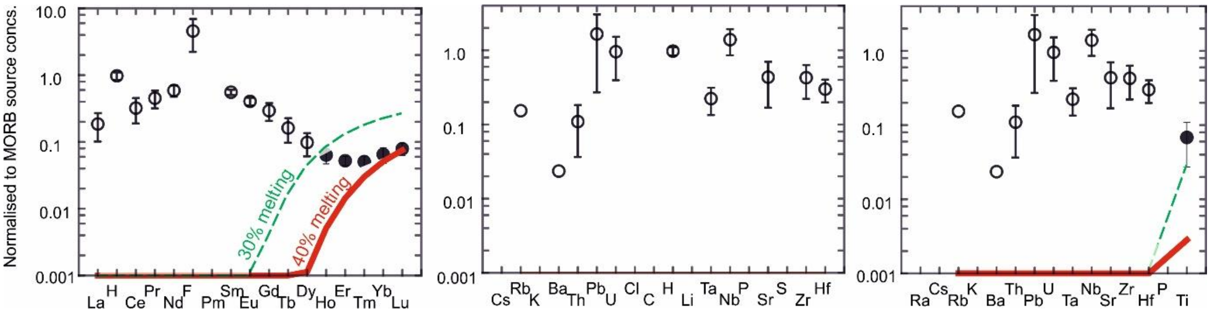

Figure S8. Rare-earth element inversion models[18,19] showing the effects of 30 and 40% melt depletion. Circles are the mean concentrations (Supplementary Data 1) and vertical bars show the standard deviaton in the orthopyroxene-rich garnet harzburgites. Predicted concentrations for 30% and 40% melting are shown by the dashed green lines and solid red lines, respectively. Open circles show elements that will be extremely depleted (stripped) from the mantle residue during 30% partial melting. Partition coefficients were calculated for a temperature of 1773 K ref[20]. MORB source normalisation values are from [17].

The large amounts of partial melting required to generate the incompatible trace element concentrations in the orthopyroxene-rich garnet harzburgites form a residue that is susceptible to modification by infiltrating melts and fluids.

Data from melt inclusions suggests that small-fraction mantle melts have higher F/Cl ratios than those associated with down-going slabs (Figure S9). Moreover, ‘shallow’ solute-poor slab derived hydrous fluids have lower F contents than ‘deeper’ solute-rich sediment melts or supercritical fluids.

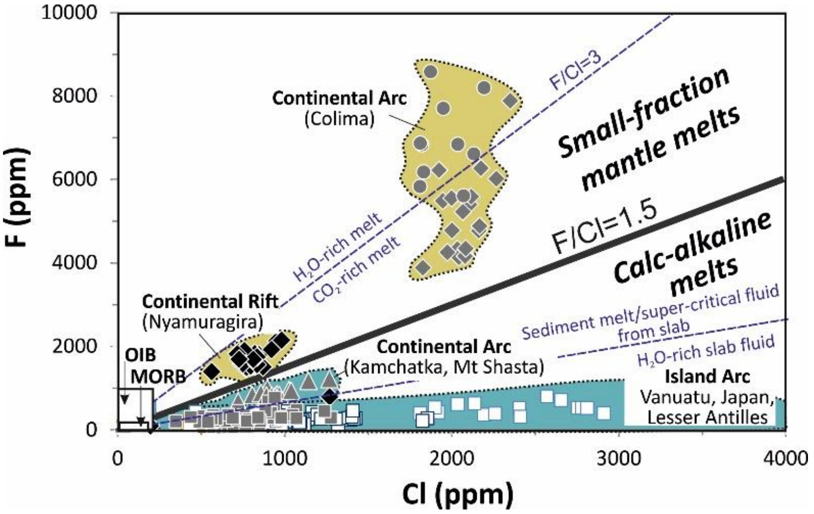

Figure S9. Variations in Cl and F contents of olivine-hosted melt inclusions found in volcanic rocks from a variety of tectonic settings. The F/Cl ratios of a range of melts and fluids are shown for comparison. Data are from [21–26].

It has recently been proposed that supercritical high-alkali fluids released during deep subduction could be significant transfer agents for Zr from the down-going slab to mantle wedge. These fluids are continuously released through the dehydration of sediments, altered oceanic crust and serpentinised lithosphere. They reach temperatures between 700°C–900°C at sub-arc depths (~80–180 km) and pressures (~2.0–6.0 GPa) and have the capacity to dissolve significant amounts of silica, alkalis, magnesium and alumina to form solute-rich supercritical fluids as they infiltrate overlying sediments and altered oceanic crust [27]. Unlike many high-density fluids (HDFs) in diamond, BD2125 has a $[Zr/Hf]_n$ that is greater than 1.5 (Figure S10) and consistent with metasomatism involving silicic low Mg carbonate supercritical fluids.

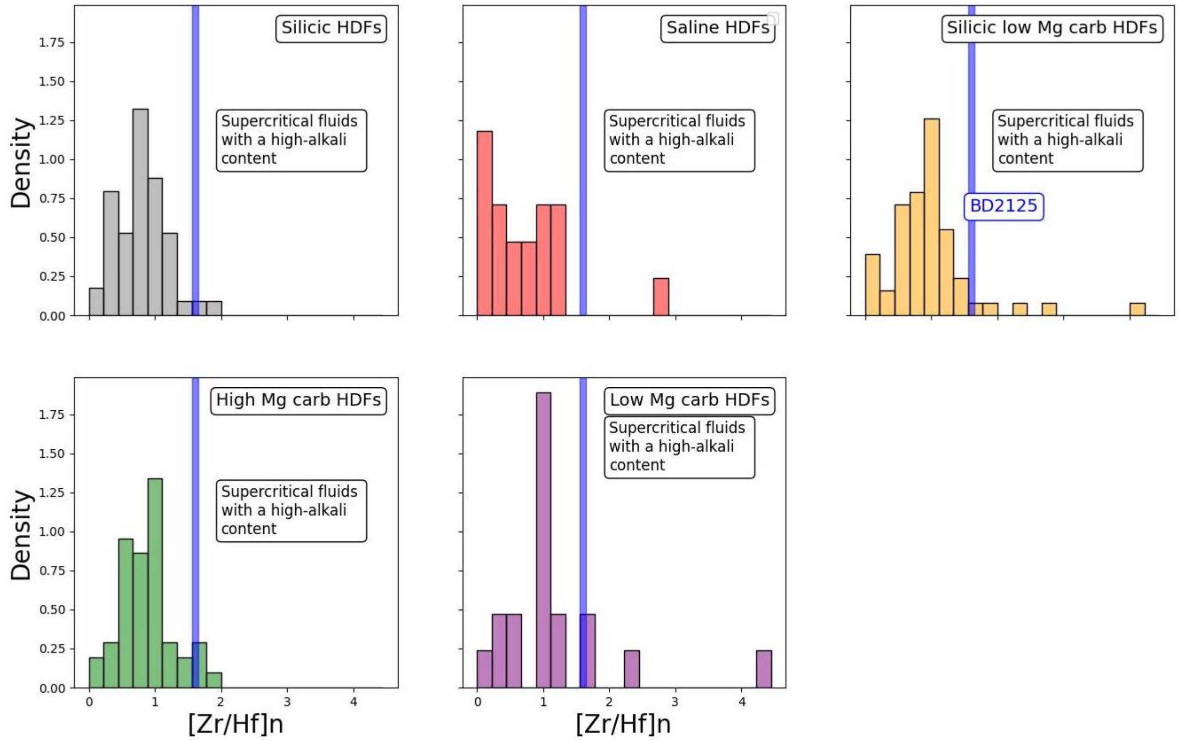

Figure S10. Frequency density plots illustrating the distribution of [Zr/Hf]n in high-density fluids (HDFs) encapsulated in diamond where n is the MORB normalised ratio from Salters & Stracke [17]. Data are from Weiss et al. [28].

Supercritical fluids derived from carbonated pelites in down-going slabs have relatively high Nb/Ta (Chen et al., 2022) and also lower Sr/Nd ratios than those derived from aqueous fluids [29]. For aqueous fluids in equilibrium with eclogite, Sr has a high partition coefficient for the fluid that increases with fluid salinity (1 to 70 but typically 10 [ref 30]; Figure S11). As a consequence eclogite fluids tend to have high $[Sr/Nd]_n$ with peaks at Sr on normalised multi-element plots [31,32]. No data are available for supercritical fluids from eclogite. Saline high-density fluids encapsulated in diamonds also have positive peaks at Sr[28]. This contrasts with silicic HDFs [28] and BD2125 which have pronounced troughs at Sr relative to elements of similar compatibility in mantle melts, e.g. Nd (Figures 5 & 9 and S11).

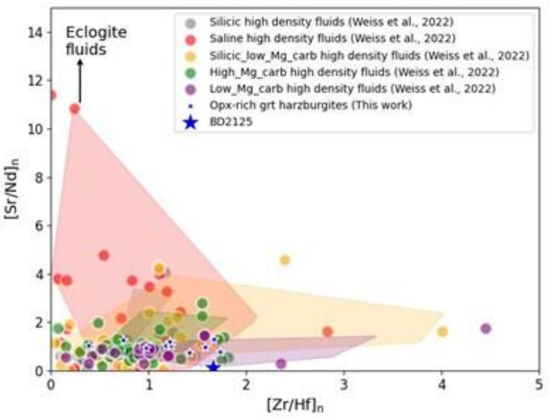

Figure S11. Variation in MORB source normalised Zr /Hf versus Sr/Nd of high density fluids in diamond inclusions [28], eclogite aqueous fluids from experiments [30] and orthopyroxene-rich garnet harzburgites (this work).

References

1. Priestley, K. & McKenzie, D. The relationship between shear wave velocity, temperature, attenuation and viscosity in the shallow part of the mantle. *Earth Planet Sci. Letts.* **381**, 78–91 (2013).
2. Giuliani, A. & Pearson, D. G. Kimberlites: From Deep Earth to Diamond Mines. *Elements* **15**, 377–380 (2019).
3. Jackson, C. J. & Gibson, S. A. Build-up of multiple volatiles in Earth's continental keels: Implications for craton stability. *Earth Planet Sci. Letts.* **611**, 118134 (2023).
4. Dawson, J. B. & Smith, J. V. Occurrence of diamond in a mica–garnet lherzolite xenolith from kimberlite. *Nature* **254**, 580–581 (1975).
5. Peslier, A. H., Woodland, A. B., Bell, D. R., Lazarov, M. & Lapen, T. J. Metasomatic control of water contents in the Kaapvaal cratonic mantle. *Geochimica et Cosmochimica Acta* **97**, 213–246 (2012).
6. Peslier, A. H., Woodland, A. B., Bell, D. R. & Lazarov, M. Olivine water contents in the continental lithosphere and the longevity of cratons. *Nature* **467**, 78–81 (2010).
7. Doucet, L. S. *et al.* High water contents in the Siberian cratonic mantle linked to metasomatism: An FTIR study of Udachnaya peridotite xenoliths. *Geochimica et Cosmochimica Acta* **137**, 159–187 (2014).
8. Aulbach, S., Griffin, W. L., Pearson, N. J., O'Reilly, S. Y. & Doyle, B. J. Lithosphere formation in the central Slave Craton (Canada): plume subcretion or lithosphere accretion? *Contrib Mineral Petrol* **154**, 409–427 (2007).
9. Kilgore, M. L. *et al.* Metasomatic control of hydrogen contents in the layered cratonic mantle lithosphere sampled by Lac de Gras xenoliths in the central Slave craton, Canada. *Geochimica et Cosmochimica Acta* **286**, 29–53 (2020).
10. Lee, C.-T. & Rudnick, R. L. Compositionally stratified cratonic lithosphere: Petrology and geochemistry of peridotite xenoliths from the Labait tuff cone, Tanzania. in *Gurney, J. J., Gurney, J. L., Pascoe, M. D. & Richardson, S. H. (eds) Proceedings Volume of 7th International Kimberlite Conference, Cape Town* 503–521 (Cape Town, 1999).
11. Branchetti, M., Zepper, J. C. O., Peters, S. T. J., Koornneef, J. M. & Davies, G. R. Multi-stage garnet formation and destruction in Kimberley harzburgitic xenoliths, South Africa. *Lithos* **390–391**, 106119 (2021).

12. Urann, B. M. *et al.* Fluorine and chlorine in mantle minerals and the halogen budget of the Earth's mantle. *Contributions to Mineralogy and Petrology* **172**, 10.1007/s00410-017-1368–7 (2017).
13. Gibson, S. A., Rooks, E., Day, J. A., Petrone, C. M. & Leat, P. T. The role of sub-continental mantle as both 'sink' and 'source' in deep Earth volatile cycles. *Geochimica et Cosmochimica Acta* (2020).
14. Withers, A. C., Bureau, H., Raepsaet, C. & Hirschmann, M. M. Calibration of infrared spectroscopy by elastic recoil detection analysis of H in synthetic olivine. *Chemical Geology* **334**, 92–98 (2012).
15. Heckel, C. *Sheared Peridotites: Linking Deformation and Metasomatism Contributing to the Onset of Craton Destabilization*. (Johann Wolfgang Goethe-Universität Frankfurt am Main, 2023).
16. Viljoen, K. S., Harris, J. W., Ivanic, T., Richardson, S. H. & Gray, K. Trace element chemistry of peridotitic garnets in diamonds from the Premier (Cullinan) and Finsch kimberlites, South Africa: Contrasting styles of mantle metasomatism. *Lithos* **208–209**, 1–15 (2014).
17. Salters, V. J. M. & Stracke, A. Composition of the depleted mantle. *Geochem. Geophys. Geosyst.* **5**, 200410.1029/2003GC000597 (2004).
18. Gibson, S. A. & Richards, M. A. Delivery of deep-sourced, volatile-rich plume material to the global ridge system. *Earth and Planetary Science Letters* **499**, 205–218 (2018).
19. McKenzie, D. & O'Nions, R. K. Partial melt distributions from inversion of rare earth element concentrations. *J. Petrology* **32**, 1021–1091 (1991).
20. McKenzie, D. Speculations on the Generation and Movement of Komatiites. *Journal of Petrology* **61**, egaa061 (2020).
21. Voyer, M. L., Rose-Koga, E. F., Shimizu, N., Grove, T. L. & Schiano, P. Two Contrasting H2O-rich Components in Primary Melt Inclusions from Mount Shasta. http://petrology.oxfordjournals.org.
22. Head, E., Shaw, A., Wallace, P., Sims, K. & Carn, S. Insight into volatile behavior at Nyamuragira Volcano (D.R. Congo, Africa) through olivine-hosted melt inclusions. *Geochemistry Geophysics Geosystems* 10.1029/2011GC003699 (2011) doi:10.1029/2011GC003699.
23. Rose-Koga, E. F. *et al.* Volatile (F and Cl) concentrations in Iwate olivine-hosted melt inclusions indicating low-temperature subduction. *Earth, Planets and Space* **66**, 81 (2014).
24. Vigouroux, N., Wallace, P. J. & Kent, A. J. R. Volatiles in High-K Magmas from the Western Trans-Mexican Volcanic Belt: Evidence for Fluid Fluxing and Extreme Enrichment of the Mantle Wedge by Subduction Processes. *J. Petrology* **49**, 1589–1618 (2008).
25. Bouvier, A.-S., Métrich, N. & Deloule, E. Slab-Derived Fluids in the Magma Sources of St. Vincent (Lesser Antilles Arc): Volatile and Light Element Imprints. *J. Petrology* **49**, 1427–1448 (2008).
26. Portnyagin, M., Hoernle, K., Plechov, P., Mironov, N. & Khubunaya, S. Constraints on mantle melting and composition and nature of slab components in volcanic arcs from volatiles (H2O, S, Cl, F) and trace elements in melt inclusions from the Kamchatka Arc. *Earth and Planetary Science Letters* **255**, 53–69 (2007).
27. Chen, W., Xiong, X. & Takahashi, E. Zircon Solubility in Solute-Rich Supercritical Fluids and Zr Transfer From Slab to Wedge in the Deep Subduction Process. *Journal of Geophysical Research: Solid Earth* **126**, e2021JB021970 (2021).
28. Weiss, Y., Czas, J. & Navon, O. Fluid Inclusions in Fibrous Diamonds. *Reviews in Mineralogy and Geochemistry* **88**, 475–532 (2022).
29. Kessel, R., Schmidt, M. W., Ulmer, P. & Pettke, T. Trace element signature of subduction-zone fluids, melts and supercritical liquids at 120-180[thinsp]km depth. *Nature* **437**, 724–727 (2005).
30. Rustioni, G., Audetat, A. & Keppler, H. The composition of subduction zone fluids and the origin of the trace element enrichment in arc magmas. *Contrib Mineral Petrol* **176**, 51 (2021).
31. Tsay, A., Zajacz, Z., Ulmer, P. & Sanchez-Valle, C. Mobility of major and trace elements in the eclogite-fluid system and element fluxes upon slab dehydration. *Geochimica et Cosmochimica Acta* **198**, 70–91 (2017).
32. Gong, T.-N. *et al.* Mineral precipitation sequence from multi-stage fluids released by eclogite during high-pressure metamorphism. *American Mineralogist* **110**, 136–153 (2025).
