# Supplementary material for: The role of C-O-H-F-Cl fluids in the making of Earth’s continental roots
Source: Nat Commun. 2025 Aug 22;16:7842. doi: 10.1038/s41467-025-62888-3 (PMC12373800; doi:10.1038/s41467-025-62888-3)
Supplement: Supplementary file 2 — Description of Additional Supplementary Information [file 41467_2025_62888_MOESM2_ESM.pdf]

Description of Additional Supplementary Information

Supplementary Data 1: Summary table providing details of the modal mineralogy, PT estimates plus major, trace and volatile analyses of mineral phases together with calculated bulk-rock concentrations.

Supplementary Data 2: LA-ICP-MS analyses of standards

Supplementary Data 3: SIMS analyses

Supplementary Data 4: EPMA analyses of phlogopite

Supplementary Data 5: Statistical analysis of volatile contents of different mantle lithologies

Supplementary Data 6: Statistical analysis of bulk volatile concentrations in mantle lithologies.
